# Hepatitis C Antibodies among Blood Donors, Senegal, 2001

**DOI:** 10.3201/eid0911.030191

**Published:** 2003-11

**Authors:** Jean-François Etard, Pierre Colbachini, Jacques-Albert Dromigny, Jean-David Perrier-Gros-Claude

**Affiliations:** * Institut de Recherche pour le Développement, Dakara, Senegal; † Hôpital Principal de Dakara, Dakar, Senegal; ‡ Institut Pasteur de Dakar, Dakar, Senegal

**To the Editor:** Prevalence of chronic hepatitis C virus (HCV) among blood donors has been assessed in a few West African countries; most recent estimates range from 1.1% to 6.7% (*1*–*4*). A recent meta-analysis of studies, including a confirmation test, yielded an average prevalence of HCV infection of 3.0% (*5*). Until 2001, no systematic screening of HCV infection occurred among blood donors in Senegal, and blood donation legislation is still pending. We report an assessment of the proportion of blood donors from the Hôpital Principal de Dakar who had HCV antibodies in 2001.

Blood donors were all volunteers, recruited independently from the hospitalized patients and registered in a local donors association. We screened for risk factors for bloodborne infections in potential donors through a clinical examination and a confidential questionnaire. Persons with a history of jaundice or a risk behavior were excluded. Serum samples collected from blood donors from June to December 2001 were screened for HCV antibodies by a third-generation enzyme immunoassay (EIA) (HCV Murex 4.0; Abbott Laboratories, Abbott, IL). Confirmation was performed by a recombinant-immunoblot assay (INNO-LIA HCV Ab III update; [Innogenetics, Gent, Belgium]). HCV RNA was detected by a qualitative reverse transcription–polymerase chain reaction (Roche Amplicor HCV test [Hoffman-LaRoche, Basel, Switzerland]). Genotype was determined by the INNO-LiPA HCV II assay (Innogenetics). Presence of hepatitis B surface antigen (HbsAg) and alanine-aminotransferase (ALAT) level are routinely assessed, as well as HIV and human T-lymphotropic virus type l infection.

The age of the 1,081 donors ranged from 18 years to 61 years (mean 35.6 years), and 81% were men. First-time donors accounted for 31% and were younger than repeat donors (mean 30.5 years vs. 37.8 years; p < 10^–4^). EIA HCV antibodies were found in 18 donors (1.6%). Immunoblot assay was positive for nine, yielding an overall prevalence of 0.8% (exact 95% confidence interval 0.4% to 1.5%). Eight of the nine were repeat donors, but the difference in prevalence compared with first-time donors did not reach statistical significance (1.1% vs. 0.3%). HCV-infected donors tended to be older than uninfected donors (mean 42.3 years vs. 35.5 years, median 46.7 years vs. 34.6 years, Mann-Whitney test p = 0.04), and the trend with age was significant (18–29 years 0.3%; 30–39 years 0.6%; 40–49 years 1.5%; >50 years 1.8%; chi-square trend = 4.39; p = 0.03). ALAT levels of infected study participants were in the normal range (17–55 IU). One participant had an ALAT level above normal. Genotype 2ac has been identified on line immunoassay–positive samples (three samples not tested). HBsAg was detected in 13% of the new donors. No co-infection with HCV and hepatitis B virus was found.

The prevalence of HCV antibodies in blood donors in Dakar in 2001 appears to be one of the lowest in West Africa, close to published estimates for Mauritania and Benin (1.1% and 1.4%, respectively) and lower than in other West African countries such as Ghana or Guinea, where prevalence ranges from 2.8% to 6.7% (*1*–*4*). This finding is in keeping with results of a hospital case-control study on HCV infection and liver cirrhosis or cancer, conducted in 1995 in Dakar. While that study did not identify HCV infection in 73 controls, 2 of 73 case-patients (2.7%) had HCV antibodies (*6*). Conversely, high HCV prevalence was found in groups at risk: antibodies were present in 12 of 15 hemodialysis patients, and HCV RNA was found in 6 of the 12 HVC antibody-positive patients (genotype 2ac, the same as in our study); 7% of a cohort of 58 HIV-1 patients receiving highly active antiretroviral therapy had a positive HCV serologic result (*7*,*8*).

In the urban setting of Dakar, HCV infection seems still to be confined to groups at risk. The contribution of HCV to chronic liver diseases has not been yet demonstrated. Approximately 15,000 blood donations are annually made in Dakar. A systematic screening of HCV antibodies in blood donors could prevent, on average, 120 bloodborne HCV infections each year. Given these data and the price of EIA and LIA, the screening cost per HCV-positive sample identified, and infection subsequently averted, is approximately 200,300 CFA (U.S.$305). This estimate is low since it includes only the marginal cost of the reagent kits. This screening cost could be reduced by discarding blood units that test positive after only one enzyme-linked immunosorbent assay (156,000 CFA or U.S.$237), at the price of nearly 3% of blood units wrongly discarded. France has demonstrated that this strategy has the best cost-effectiveness ratio, as long as the prevalence remains below 8% (*9*). This cost compares favorably with the cost per HIV infection averted through improvement of blood safety (range U.S.$20–U.S.$1,000), assessed in some highly HIV-prevalent southern African countries (Tanzania, Zambia, Zimbabwe) (*10*). The HCV-positive discarded blood units will be added to the blood units testing positive for hepatitis B surface (13%), HIV, and HTLV, which accounted for nearly one third of all donations in 2001. These findings argue in favor of maintaining a roster of regular, seronegative donors to save numbers of blood units.
